# Swedish civil air traffic control dataset

**DOI:** 10.1016/j.dib.2023.109240

**Published:** 2023-05-16

**Authors:** Jens Nilsson, Jonas Unger

**Affiliations:** a Linköping University, Department of Science and Technology, Linköping, 581 83, Sweden and LFV, Technical Department, Norrköping, 601 79, Sweden; b Linköping University, Department of Science and Technology, Linköping, 581 83, Sweden

**Keywords:** Air transport, Flight tracks, Airspace data, GRIB weather data

## Abstract

The Swedish Civil Air Traffic Control (SCAT) dataset consists of 13 weeks of data collected from the area control in Sweden flight information region. The dataset consists of detailed data from almost 170,000 flights as well as airspace data and weather forecasts. The flight data includes system updated flight plans, clearances from air traffic control, surveillance data and trajectory prediction data. Each week of data is continuous but the 13 weeks are spread over one year to provide variations in weather and seasonal traffic patterns. The dataset does only include scheduled flights not involved in any incident reports. Sensitive data such as military and private flight has been removed.

The SCAT dataset can be useful for any research related to air traffic control, e.g. analysis of transportation patterns, environmental impact, optimization and automation/AI.

**Specifications Table**

**Table:** 

Subject	Engineering: Aerospace Engineering
Specific subject area	The dataset consists of detailed data of almost 170,000 flights, weather forecasts and airspace data from the Swedish air traffic control system.
Type of data	JSON files Tables (for the data format specification)
How the data were acquired	The data was acquired by extracting information from the Swedish air traffic control systems.
Data format	Transformed Filtered
Description of data collection	The dataset includes surveillance data, air traffic controller input, flight planning, trajectory prediction, airspace and weather data from Swedish area control.
Data source location	Institution: LFV (Swedish air navigation service provider) Country: Sweden (Swedish Flight Information Region)All the data in this dataset originates from data recorded by the TopSky air traffic control system [1], however some of the data in TopSky originates from other sources: • Surveillance data originates from the ARTAS system [2] which is a multi-sensor tracker, which gets its data from radar stations and wide area multilateration sensors in Sweden. • Weather forecasts originates from the world meteorological organization [3] in London. • Flight planning data originates from the flight plans submitted by pilots/airline operators in their respective countries, sent via the aeronautical networks AFTN/AMHS to TopSky. Flight plan updates are also sent from neighboring flight information regions using the OLDI protocol. • Airspace data mostly originates from Aeronautical Information Publications (AIP) made in relevant countries, but alterations and addition may be done to adapt the information to TopSky. Data that originates from these other systems may have been modified by TopSky before being recorded.
Data accessibility	Repository name: Mendeley data Data identification number: DOI: 10.17632/8yn985bwz5.1 Direct URL to data: https://data.mendeley.com/datasets/8yn985bwz5

## Value of the Data

• A key challenge in the research of air traffic control is that there is a lack of publicly available data [4,5]. The main reasons is that data in this domain in many cases is classified and/or rely on proprietary software systems and data formats.
• This dataset is unique in its kind. Other publicly available sources of air traffic related data exists, e.g. OpenSky Network [6] and ADS-B Exchange [7], however these sources are limited to ADS-B data and are as such lacking the comprehensive coverage of the full information related to each flight as presented in the this data set.
• This dataset can be useful for any research related to air traffic and air traffic control, e.g. automation and support tools for air traffic control, environmental research and airspace optimization.

## 1 Objective

There is currently a lack of high-quality open datasets for research around air traffic control and air transportation. The main objective behind the SCAT dataset [8] is to enable in-depth analysis and research in the context of aviation. We foresee that SCAT can be used in the research and development of future AI and machine learning based tools for air traffic control.

## 2 Data Description

The Swedish Civil Air Traffic Control (SCAT) dataset [8] contains detailed data of almost 170,000 flights, weather forecasts and airspace data from a perspective of air traffic control. The data originates from the air traffic control systems at the two control centers, Malmö (ESMM) and Stockholm (ESOS), which provide upper area control in the Swedish flight information region (FIR). The data is organized in 13 compressed archives in ZIP format, each containing one week of continuous data, see Table 1. The data has been filtered and processed as described in the “Experimental design, materials and methods” section.

**Table 1:** The files and data periods in the SCAT dataset.

File name	Start date	End date	Number of flights
scat20161015_20161021.zip	2016-10-15	2016-10-21	13,138
scat20161112_20161118.zip	2016-11-12	2016-11-18	12,248
scat20161210_20161216.zip	2016-12-10	2016-12-16	12,099
scat20170107_20170113.zip	2017-01-07	2017-01-13	11,195
scat20170215_20170221.zip	2017-02-15	2017-02-21	11,610
scat20170304_20170310.zip	2017-03-04	2017-03-10	12,265
scat20170401_20170407.zip	2017-04-01	2017-04-07	13,255
scat20170429_20170505.zip	2017-04-29	2017-05-05	12,855
scat20170527_20170602.zip	2017-05-27	2017-06-02	13,832
scat20170624_20170630.zip	2017-06-24	2017-06-30	13,772
scat20170722_20170728.zip	2017-07-22	2017-07-28	12,950
scat20170819_20170825.zip	2017-08-19	2017-08-25	13,963
scat20170916_20170922.zip	2017-09-16	2017-09-22	14,365
		**Total flights**	**167,547**

All files inside the archives are in the JavaScript Object Notation (JSON) format [9]. Time stamps are in UTC time and represented as strings in ISO 8601 format without explicit time zone (e.g. 2017-06-06T13:45:10.362). Properties without values may be null or left out, depending on the source for the data. To reduce the number of tables needed to document each object type, several object types may be represented in one table. In such case indentation and the sign • is used in front of the property name to indicate that it is a property of the object at the previous indentation level.

Each archive contains several flight files, which are files named with the unique id number given to the flight during data extraction, e.g. *101234.json*. Each file holds all the data related to a single flight such as:

• The sequence of control centers controlling the flight.
• Data related to flight planning, coordination and clearances from the air traffic controllers.
• Surveillance data from the system tracker which process' the information from multiple radar and wide area multilateration sources into a single traffic view.
• Data from the trajectory prediction subsystem in TopSky which repeatedly makes updated predictions of the future flight trajectory.

**Fig. 1:**
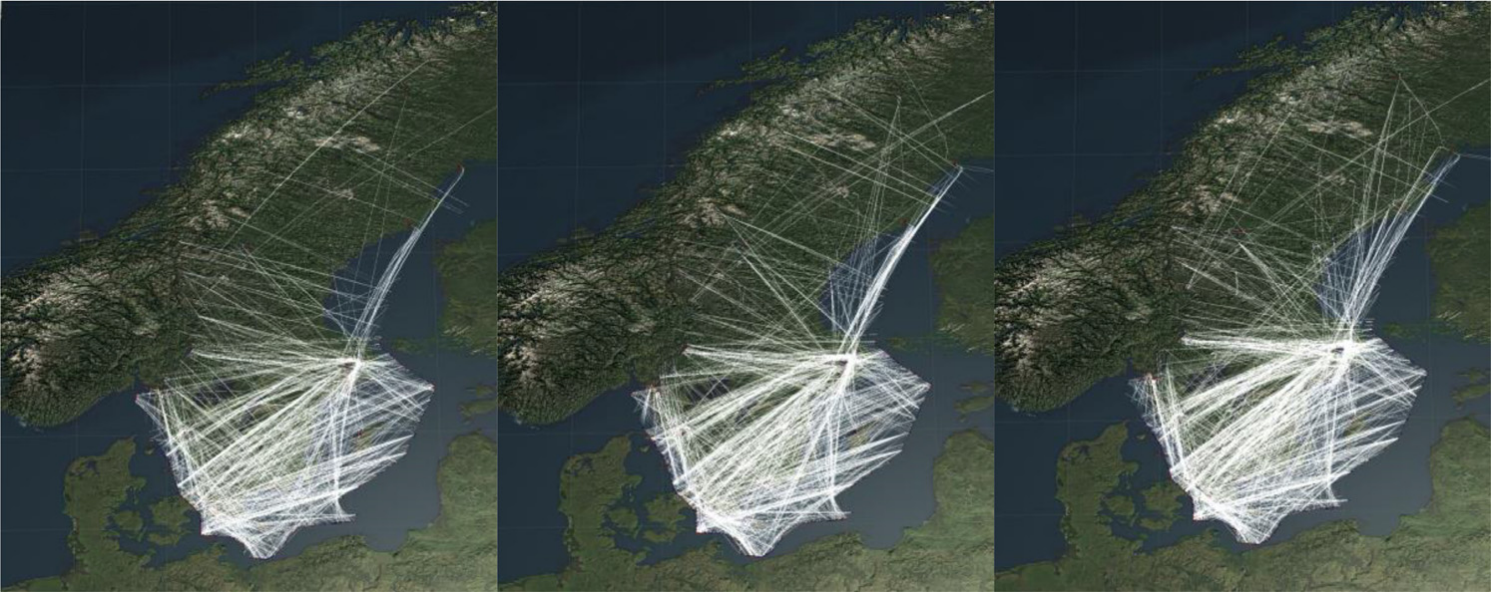
Visualizations of the surveillance data showing the flights in Swedish airspace on three different days. The images are captured from our flight information visualization tool developed using the Unity3D platform. From left to right; 016–11–12 (Sunday) 1160 Flights, 2016–11–13 (Monday) 1575 Flights, 2016–11–14 (Tuesday) 1915 Flights.

The format of the top level object in the flight files is described in Table 2 and its contained types are described in Table 3, Table 4, Table 5, Table 6, Table 7, Table 8, Table 9, Table 10, Table 11. Fig. 1 shows three example visualizations where the trajectories of flights in Swedish airspace from different dates are illustrated.

**Table 2:** The top properties of a Flight object.

property name	type	description
center_ctrl	[object]	array of control center objects sorted by *start_time*.
• center_id	number	the control center unique id: 1 = esmm 2 = esos
• start_time	string	The time when the control centers is deemed the most relevant data source for the current flight.
Fpl	object	Flight plan related information, see Table 3.
Id	number	Integral number with the flights unique id (corresponds to the file name).
Plots	[object]	Array of plots from surveillance system, see Table 10.
predicted_trajectory	[object]	Trajectory predictions from air traffic control system, see Table 11

**Table 3:** The properties of the flight plan object.

Property name	Type	Description
fpl_arr	[object]	Array of flight arrival information sorted by *time_stamp*, see Table 4.
fpl_base	[object]	Array of basic flight plan information sorted by *time_stamp*, see Table 5.
fpl_clearance	[object]	Array of given clearances sorted by *time_stamp*, see Table 6.
fpl_dep	[object]	Array of flight departure information sorted by *time_stamp*, see Table 7.
fpl_holding	[object]	Array of holding information sorted by *time_stamp*, see Table 8.
fpl_plan_update	[object]	Array of flight plan updates sorted by *time_stamp*, see Table 9.

**Table 4:** The properties of flight plan arrival objects.

Property name	Type	Description
approach_clearance	bool	True if the aircraft has been cleared for approach, false otherwise.
arrival_runway	string	Name of the runway the aircraft will land on.
Ata	string	Actual time of arrival.
missed_approach_flag	bool	Set to true if a missed approach has occurred.
Star	string	Name of the assigned STAR (Standard arrival route) if any.
time_stamp	string	Time stamp of when the information was updated.

**Table 5:** The properties of a basic flight plan information object.

Property name	Type	Description
Adar	string	Actual destination aerodrome if different from ades (ICAO code).
Adep	string	Departure aerodrome (ICAO code).
Ades	string	Destination aerodrome (ICAO code).
aircraft_type	string	Aircraft type (ICAO code).
Callsign	string	Aircraft callsign (ICAO code).
equip_status_rvsm	bool	True if aircraft is equipped to fly in RVSM (Reduced Vertical Separation Minima) airspace.
flight_rules	string	Flight rules from the filed flight plan item 8.
time_stamp	string	Time stamp when the information was updated.
Wtc	string	Wake turbulence category from the filed flight plan item 9.

**Table 6:** The properties of a flight plan clearances object.

Property name	Type	Description
assign_heading_beacon	string	If the aircraft is assigned a heading towards a beacon or navigation point, this field is set to the name of that point otherwise it is null.
assigned_heading_val	number	Assigned heading in degrees or null if no heading is assigned.
assigned_speed_unit	string	Unit of the assigned speed: ``KNOT'' = nautical miles per hour ``MACH'' = mach number ``KMHOUR'' = kilometers per hour or null if no speed is assigned.
assigned_speed_val	number	Assigned speed value, with unit according to the field *assigned_speed_unit*, or null if no speed is assigned.
Cfl	number	Cleared flight level/altitude as specified in *cfl_unit* or null if lacking clearance.
cfl_unit	string	The unit of the cleared flight level: ``A'' = altitude in feet ``F'' = flight level.
time_stamp	string	Time stamp when the information was updated.

**Table 7:** The properties of a flight plan clearances object.

Property name	Type	Description
Atd	string	Actual time of departure.
departure_runway	string	Departure runway.
Iobt	string	Initial of block time.
Sid	string	Standard Instrument Departure Route.
time_stamp	string	Time stamp when the information was updated.

**Table 8:** The properties of a flight plan holding object.

Property name	Type	Description
hold_stack_vol_name	string	Name of the holding if applicable.
holding_entry_time	string	Estimated time when entering holding.
holding_leaving_time	string	Estimated time when entering holding.
holding_stack_status_id	string	Holding stack status, either: ``APPROACHING HOLD'' ``HOLD'' ``LEAVING HOLD'' ``NO HOLD''
holding_status_id	string	Holding status, either: ``HOLD ON FIX'' ``HOLD ON POSITION'' ``NO HOLD'' ``INIT HOLD'' ``HOLD ON VOLUME''
time_stamp	string	Time stamp when the information was updated.

**Table 9:** The properties of a flight plan update object.

Property name	Type	Description
copn	string	Coordination entry point name.
copn_pel	number	Planed entry level at the point specified in *copn*.
copn_pel_unit	string	The unit of altitude at the *copn_pel*: ``A'' = altitude in feet ``F'' = flight level in 100 feet
copx	string	Coordination exit point name.
copx_pel	number	Planed exit level at the point specified in copx.
copx_pel_unit	string	The unit of altitude at the *copx_pel* ``A'' = altitude in feet ``F'' = flight level in 100 feet
icao_route	string	Current route according to format in ICAO flight plan item 15.
rfl_string	string	Requested flight level according to format in ICAO flight plan item 15.
tas_string	string	Requested speed according to format in ICAO flight plan item 15.
time_stamp	string	Time stamp when the information was updated.

**Table 10:** The properties of a plot object. This data is converted from Asterix cat 62 [11], and the property names corresponds to the name in the Asterix specification.

Property name	Type	Description
time_of_track	string	Time stamp plot was updated.
I062/105	object	Calculated WGS-84 track position.
• lat	number	Latitude in WGS-84 coordinates.
• lon	number	Longitude in WGS-84 coordinates.
I062/136	object	Measured flight level.
• measured_flight_level	string	Altitude at standard air pressure in units of 100 feet.
I062/185	object	Calculated Cartesian track velocity.
• vx	number	Speed in m/s (positive is geographical east).
• vy	number	Speed in m/s (positive is geographical north).
I062/200	object	Mode of movement.
• adf	bool	Altitude discrepancy, *true* if discrepancy is detected.
• long	number	Longitudinal ground speed acceleration: 0 = Constant, 1 = Increasing, 2 = Decreasing, 3 = Undetermined.
• trans	number	Transversal acceleration: 0 = Constant course, 1 = Right turn, 2 = Left turn, 3 = Undetermined.
• vert	number	Vertical rate: 0 = Level, 1 = Climb, 2 = Descent, 3 = Undetermined.
I062/220	object	Calculated rate of climb/descent.
• rocd	number	Vertical in feet/minute (negative values are descent).
I062/380	object	Aircraft derived data.
• subitem3	object	Magnetic heading.
• ag_hdg	number	Magnetic heading in degrees.
• subitem6	object	Selected altitude. From either the FMS, the Altitude Control Panel, or the current aircraft altitude.
• altitude	number	Selected altitude in feet.
• sas	bool	Source information provided, true if *source* contains valid information.
• source	number	Source: 0 = Unknown, 1 = Aircraft altitude, 2 = FCU/MCP altitude, 3 = FMS altitude.
• subitem7	object	Final state selected altitude. The vertical intent value that corresponds with the ATC cleared altitude, as derived from the Altitude Control Panel (FCU/MCP).
• ah	bool	Altitude hold active.
• altitude	number	Selected altitude in feet.
• am	bool	Approach mode active.
• mv	bool	Managed vertical mode active.
• subitem13	object	Barometric vertical rate.
• baro_vert_rate	number	Barometric rate of climb/descent in feet per minute (negative values indicates descent).
• subitem26	object	Indicated airspeed.
• ias	number	Indicated air speed in knots.
• subitem27	object	Mach number.
• mach	number	Mach number.

**Table 11:** The properties of a predicted trajectory object.

Property name	Type	Description
route	[object]	Array of predicted route points.
• afl_unit	string	Unit for estimated flight level at point: ``A'' = altitude in feet ``F'' = flight level in 100 feet
• afl_value	number	Value for estimated flight level at point.
• eto	string	Estimated time over point.
• fix_kind	string	Fix kind is a short text from the system describing the type of point.
• fix_name	string	Name of fix, if it is a named point, or coordinates as a string in degrees and minutes.
• is_ato	bool	True if the aircraft has passed this point, false otherwise.
• lat	number	Latitude in WGS-84 coordinates.
• lon	number	Longitude in WGS-84 coordinates.
• point_to_be_used_as_cop	bool	True if the point is to be used as sector coordination point.
• rfl_unit	string	Unit of requested flight level at this point: ``A'' = altitude in feet ``F'' = flight level in 100 feet
• rfl_value	number	Value of the requested flight level at this point.
• rule	string	Flight rules at this point, format as in flight plan item 8.
time_stamp	string	Time when the route was predicted.

In each archive there is also one file named *airspace.json* that contains coordinates for all named points as well as all the extents of the control sectors for each of the centers. The format of the airspace file is described in Table 12. The airspace is valid for the entire week of data since the dates was chosen such that there were no configuration changes.

**Table 12:** The properties of the airspace file.

Property name	Type	Description
*unnamed*	[object]	array of airspace information objects.
• center_id	string	the control center unique id: ``1'' = esmm ``2'' = esos
• name	string	Name of the control center.
• points	[object]	Array of navigation point objects.
• lat	number	Latitude in WGS-84 coordinates.
• lon	number	Longitude in WGS-84 coordinates.
• name	string	Name of point.
• sectors	[object]	Array of air traffic control sectors.
• name	string	Name of sector.
• volumes	[object]	Array of the volumes the sector consists of.
• coordinates	[object]	Array of coordinates of the lateral boundary of the volume.
• lat	number	Latitude in WGS-84 coordinates.
• lon	number	Longitude in WGS-84 coordinates.
• max_alt	number	The maximum altitude of the volumes extent.
• min_alt	number	The minimum altitude of the volumes extent.

Finally, each archive contains one file named *grib_meteo.json*. The contents in this file are wind and temperature predictions used by the air traffic control system. This data originates from World Meteorological Organization (WMO) in London. Predictions are made every third hour for each cell in the grid. Each cell is 1.25° in size in both latitude and longitude direction and divided into 13 height bands from flight level 50 (5000 ft) to flight level 530 (53,000 ft), see Table 13. The data is in the form of an array sorted by time, longitude, latitude and altitude.

**Table 13:** The properties of the weather file.

Property name	Type	Description
*unnamed*	[object]	Array of weather prediction objects.
• alt	number	Altitude in flight levels (100 ft).
• lat	number	Latitude in WGS-84 coordinates.
• lon	number	Array of navigation point objects.
• temp	number	Temperature in degrees Celsius.
• time	string	Time stamp.
• wind_dir	number	Predicted wind direction.
• wind_spd	number	Predicted wind speed in knots.

Example of code to use this dataset is made available on GitHub [10]. At the time of writing there are three examples, one tool to index the flights contained in each zip archive and two tools to convert the data into Keyhole Markup Language (KML) for visualization.

## 3 Experimental Design, Materials and Methods

### 3.1 Data Sources

The data in the SCAT dataset originate from the air traffic control system TopSky [1], used for area control in Swedish airspace. TopSky continuously records various system data and technical logs, and stores it for up to three months. To store data for longer periods LFV uses KOOPA, a system developed in-house, that collects and stores the data in its original proprietary raw format. KOOPA also parses and stores the most commonly used data in a database to make it more accessible. Most data in the this dataset was extracted from this database except for trajectory predictions, weather data and some additional fields in the surveillance data that was extracted from the raw data. Since there are no standard formats suitable for the data in this dataset it was transformed into JSON.

### 3.2 Data Selection

The data was extracted in continuous one week time-blocks to capture the variation between weekdays as well as variations due to the time of day. To capture seasonal variations in weather conditions and traffic flow, the extracted weeks were e spread as evenly as possible over one year, see Table 1. The time periods were select with the following limitations in mind:

• To get a representative traffic sample we avoided to sample data from any time period with events that had a major impact on the traffic patterns, such as ash clouds from volcanoes or pandemics.
• To keep the data consistent we avoided any year with an update of the air traffic control systems that changed the format and/or content of the data.
• Any weeks with system configuration changes or system downtime due to maintenance were avoided in order to get continuous data with a single airspace configuration.

## 4 Data Extraction and Processing

For each selected week, all flight plans and radar plots, for public flights (see Data filtration below) passing Swedish airspace, were extracted from the KOOPA database using the individual flight plan identity code (IFPLID) as a unique identifier. Since both centers (ESMM and ESOS) track information on flights outside their respective control area many flights were represented in the data from both centers. In order to avoid duplication of information for each flight with various levels of completeness and correctness, only the data from the most relevant center were kept at all points in time. For flights controlled by both centers, a transition time was calculated as the average time of when control was released from the first center to the time when control was assumed by the second center. Data with time stamps before the transition time were then extracted from the first center in control, and the data with time stamps after the transition time from the second center. The average time was selected as a reasonable time stamp for hand-over for flights not traveling directly from the first center to the other, e.g., for example flights passing through uncontrolled or foreign airspace in between the centers. Trajectory prediction data and additional surveillance data (I062/380 Aircraft derived data) for each flight were then extracted from the raw data in KOOPA. As a last step airspace data and weather data were extracted.

### 4.1 Data Filtration

Due to regulations, LFV may only publish data on scheduled commercial flights not involved in any investigation or emergency, and the data in this dataset have been filtered accordingly. For example military and other state flights as well as general aviation (private flights) have been removed. Any publication of surveillance data (radar plots) outside of Swedish airspace are also prohibited and were therefore filtered out. A small number of the remaining flights were removed for other reasons. Flights missing an IFPLID were removed since this information is required in order to correlate flights between the two centers. Flights crossing the boundary between ESMM and ESOS more than once were removed since manual efforts would be required to sort out the most relevant data for each data type. Finally, flights that had a radar track of less than 30 s in Swedish airspace were removed since they were not regarded as useful.

### 4.2 Data Validation

As a first step, this dataset was validated during the extraction by logging values and comparing to the expected boundaries for applicable fields, out of bounds values were manually compared with the content in the original data. After the extraction, the data were compared to the content in the KOOPA database using a separate software that loaded each JSON file and compared its content with the database. Manual validation was performed on 100 randomly selected flights from each week of data, in total 1300 flights, by converting the data to KML and visually inspecting the content using Google Earth. As final step of validation we have developed a visualization tool in which we load the data and can visualize its different properties. Using the visualization tool the structure and integrity of the dataset has been validated by ocular inspection and filtering such that different aspects of the data can be checked for inconsistencies and errors. The data collected by KOOPA is validated by LFV as part of the normal system maintenance.

Even though this dataset has been subjected to extensive validation, it is important to realize that the original raw data is not free from errors. For example there are sometimes errors in the flight plans that are corrected by the air traffic controllers if and when they are detected. Air traffic controllers may make mistakes when entering values into the system or use the system in such a way that a clearance does not correspond to what actually is happening. Pilots also make mistakes and do not always not fly according to given clearances. No effort was made to identify, filter out or correct any such errors in this dataset since it is a part of normal operation, and removal of such errors would impede the analysis of realistic scenarios.

## CRediT authorship contribution statement

**Jens Nilsson:** Conceptualization, Methodology, Software, Validation, Writing – original draft. **Jonas Unger:** Conceptualization, Methodology, Supervision, Writing – review & editing.

## Declaration of Competing Interest

The authors declare that they have no known competing financial interests or personal relationships that could have appeared to influence the work reported in this paper.

## Data Availability

SCAT dataset (Original data) (Mendeley Data).
